# Melanonychia associated with subungual follicular inclusions: report of three cases^☆^^☆☆^

**DOI:** 10.1016/j.abd.2019.03.009

**Published:** 2020-02-12

**Authors:** Marina Câmara de Oliveira, Carlos Baptista Barcaui, Elisa de Oliveira Barcaui, Juan Piñeiro-Maceira

**Affiliations:** a Dermatology Service, Hospital Universitário Pedro Ernesto, Universidade do Estado do Rio de Janeiro, Rio de Janeiro, RJ, Brazil; b Department of Radiology, Faculdade de Medicina, Universidade Federal do Rio de Janeiro, Rio de Janeiro, RJ, Brazil

**Keywords:** Dermoscopy, Histology, Nails, Ultrasonography

## Abstract

Melanonychia is the change in the coloration of the nail plate resulting from the deposition of melanin. Among its causes are melanocytic hyperplasia, melanocytic activation and nail melanoma. Subungual follicular inclusions are histological findings of unknown etiology, possibly related to trauma. We present three cases of melanonychia of different etiologies with subungual follicular inclusions, an association that has not been well described and with an indefinite pathogenesis.

Melanonychia is the change in coloration of the nail plate, which varies from brown to black, due to the deposition of melanin. Its prevalence is approximately 1%, depending on age and ethnicity.^1^ The most common etiologies are benign melanocytic hyperplasia (lentigo and melanocytic nevus), melanocytic activation (racial or drug-induced melanonychia) and subungual melanoma. From the clinical point of view, it can be total or longitudinal (striated). It should be better investigated when it affects only one digit in adults, because in this case melanoma is the main differential diagnosis.^1^, ^2^

Three cases of melanonychia of distinct etiologies are reported, in which histopathology demonstrated an association with subungual follicular inclusions.

These patients sought dermatological care due to changes in nail color and were submitted to dermoscopy (DermLite Photo Polarized Light Dermatoscope DL3, 3Gen, Dana Point, USA) and high-frequency ultrasound (HFUS), 22 MHz (My Lab Touch, Esaote, Genoa, Itátia) showed hyperechogenic areas located in the nail bed (Fig. 1). Subsequently, the lesions were excised by shave and the material sent for histopathological examination.

**Figure 1:**
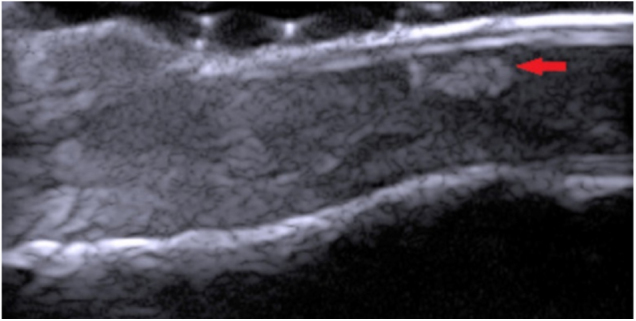
HFUS, 22 MHz, longitudinal view (Case 3). Hyperechogenic, heterogeneous area with irregular shape (arrow), avascular, located in the nail bed with Doppler mapping.

Case 1: Male, 63 years, phototype III, presenting a longitudinal melanoníquia measuring 3 mm in width, in the central portion of the left hallux, with evolution of 8 months. Irregular bands of bluish gray and light brown pigmentation were observed on dermoscopy of the nail plate, affecting the entire length of the nail until its free edge. In the histopathology subungual follicular inclusions were identified in association with melanonychia, without concomitant melanocytic lesion (Fig. 2).

**Figure 2:**
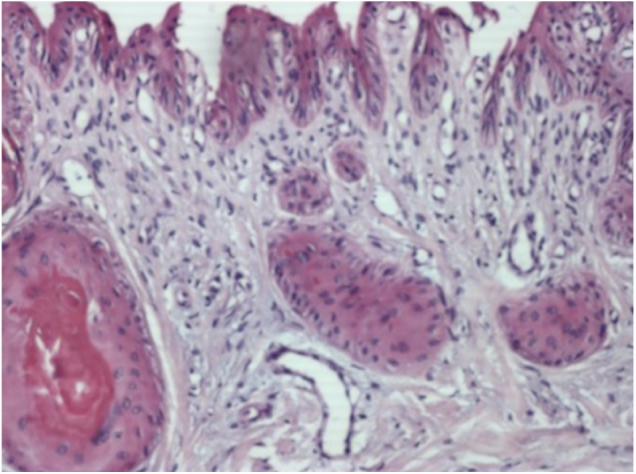
Histopathology (Case 1). Epithelial islets with keratinization of their central portion in variable degree, located in the connective tissue of the nail bed (Hematoxylin & eosin, ×400).

Case 2: Male, 43 years, phototype III, presenting a longitudinal melanoníquia measuring 2 mm in width, in the central portion in the first left quirodactyl, with a 3-month evolution. The pigmented band did not affect the distal edge of the nail (Fig. 3). Shave was performed on the nail matrix (Fig. 4) and histopathology revealed melanocytic nevus in association with follicular inclusions (Fig. 5).

**Figure 3:**
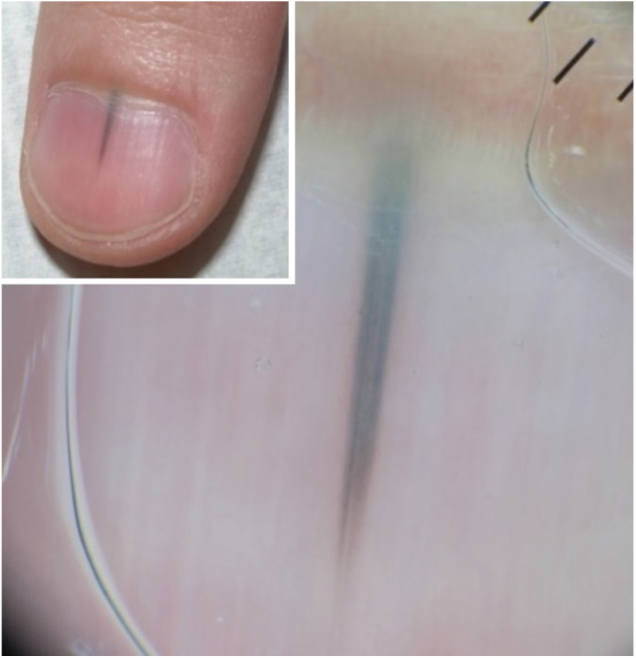
Clinic and dermoscopy (Case 2). Dermoscopy of the nail plate shows irregular bands of triangular-shaped pigmentation with a bluish-gray color and wider in the proximal and dark brown and finer portions in the distal portion.

**Figure 4:**
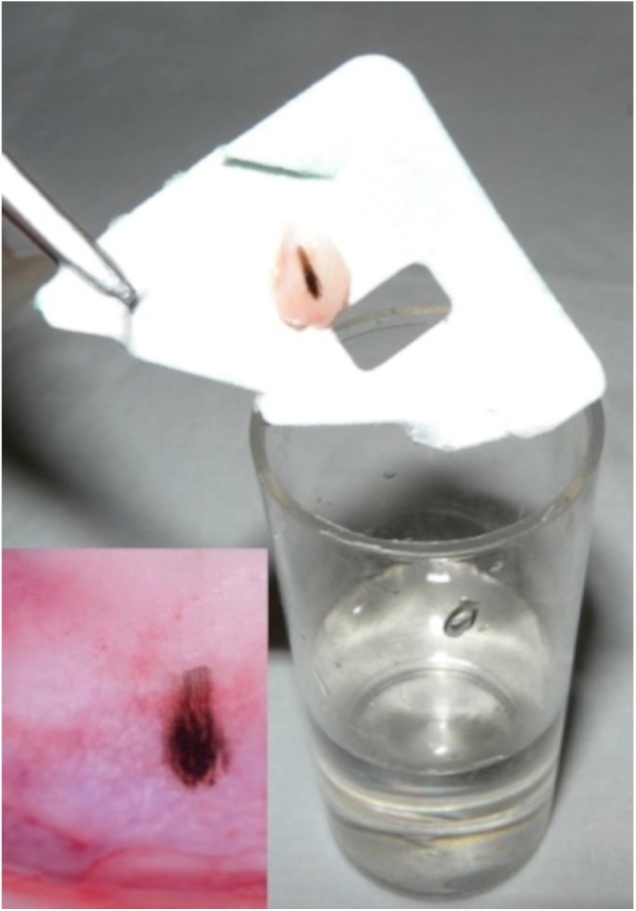
Material obtained by shaving, and dermoscopy of the nail matrix (Case 2). After shaving, the material is supported on paper before being placed in the formalin. In the nail matrix there is a small area with irregular bands of dark brown pigmentation and globules in the proximal portion.

**Figure 5:**
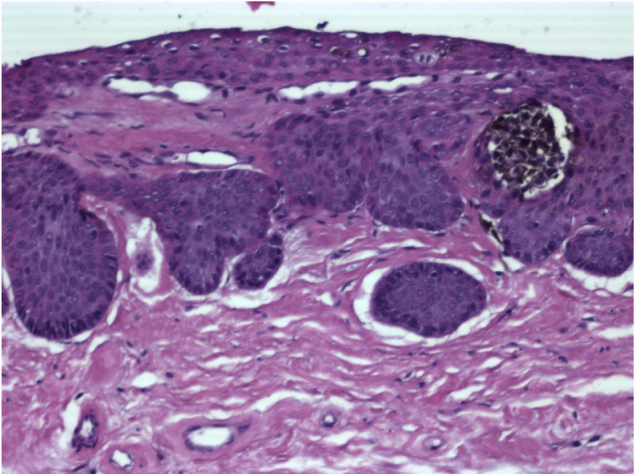
Histopathology (Case 2). Junctional nest of pigmented melanocytic cells in relation to epithelial islets located in the superficial connective tissue of the nail bed (Hematoxylin & eosin, ×400).

Case 3: Male, 53 years, phototype III, presenting a longitudinal melanonychia affecting the entire length of the right hallux, with an evolution of 2 years. Irregular bands of light brown and dark pigmentation were observed on dermoscopy of the nail plate, affecting the entire length of the nail until its free border, with pigment leakage at the proximal border (Hutchinson's sign). Histopathology revealed intraepithelial melanocytic dysplasia in association with follicular inclusions.

Subungual follicular inclusions were described by Samman in 1959, involving the toes and, in 1969, the quirodactyls by Lewin.^3^, ^4^ Since then, it has been described with different denominations, such as subungual epidermoid cysts, microcysts follicles of the nail bed, subungual calcified inclusions, among others.^5^

Although the pathogenesis is unknown, the hypothesis is that trauma is the main triggering factor, although clinical signs of trauma are not always present.^4^, ^6^, ^7^ Some authors believe that there is proliferation of dermal fibroblasts in the nail bed, consequently leading to sequestration of the bed epithelium in the dermis with consequent formation of the inclusions.^4^, ^7^

Perrin claims for the use of the term follicular microcysts for these bulbous proliferations of the epithelial cones, located in the subungual connective tissue, usually without connection to the nail bed epithelium.^**8**^ His argument is based on findings from the literature which indicate that the nail bed epithelium has vestigial follicular units, which is justified by the embryological data that the nail bed epithelium is an invagination of the dorsal epidermis that covers the finger and contains rare sprouts, unlike palms and plants. We adopted the nomenclature subungual follicular inclusions, since it includes the argument of the follicular origin proposed by Perrin^8^ and maintains part of the original nomenclature proposed by Samman and Lewin^3^, ^4^ because there is not always a microcystic formation in the epithelial inclusions.

The hallux is the most affected finger, corroborating with the hypothesis that trauma is the triggering factor.^5^, ^6^ There is no specific clinical lesion of the follicular inclusions, and it may associated itself as a nail clubbing, striations, onycholysis, subungual hyperkeratosis, anonychnia, or no apparent clinical manifestation.^5^, ^7^ When clinical changes occur, they are usually asymptomatic or, depending on the size of the inclusions, may cause nail bed edema.^4^, ^9^

Another clinical manifestation is melanonychia, the longitudinal one being the most commonly found presentation. Albeit there are no reports to confirm the hypothesis, it is suggested that it is due to the activation of the melanocytes of the nail bed.^8^ In case 2 an associated junctional nevus was observed and in case 3 there was a melanocytic dysplasia which is opposed to the theory of simple melanocytic activation. However, in case 1 histopathology did not reveal melanocytic lesion concomitant with subungual follicular inclusions.

Some tumors located in the nail bed represent a clinical differential diagnosis, such as keratoacanthoma, squamous cell carcinoma, subungual myxoid cyst and glomus tumor.^4^ The association of subungual melanoma with follicular inclusions has been reported, but the relation between them has not yet been established.^5^

The histopathological analysis of the nail bed is vital for the diagnosis. Obtaining the tissue for examination is performed after avulsion of the nail plate and biopsy. The histopathological diagnosis is based on the finding of islets of epithelium located in connective tissue, in variable number and size, with partial or complete keratinization of its central portion. These islets are not related to the nail bed epithelium.^5^, ^7^

Dermoscopy revealed an “eclipse sign”, a well-demarcated ring-shaped image that may represent the epithelial islets with central keratinization.^9^ This finding is useful to differentiate from other nail tumors, but was not found in any of reported cases.

The subungual follicular inclusions can be observed in HFUS as hypo or hyperechogenic areas with irregular shape, located in the nail bed, hypoechoic. In Doppler mapping, the structure is avascular, which differentiates itself from tumors of the nail unit.^10^

Subungual follicular inclusions are proliferative, epithelial, benign lesions, and often incisional biopsy is both diagnostic and therapeutic.^7^

Since the subungual follicular inclusions are histopathological findings poorly described in the literature, with nine published articles, it is important to know more about this condition, since it can cause different clinical manifestations in the nails.

## Financial support

None declared.

## Authors’ contributions

Marina Câmara de Oliveira: Approval of the final version of the manuscript; conception and planning of the study; elaboration and writing of the manuscript; obtaining, analysis, and interpretation of the data; effective participation in research orientation;

Carlos Baptista Barcaui: Approval of the final version of the manuscript; conception and planning of the study; elaboration and writing of the manuscript; obtaining, analysis, and interpretation of the data; effective participation in research orientation; intellectual participation in the propaedeutic and/or therapeutic conduct of the studied cases; critical review of the literature; critical review of the manuscript.

Elisa de Oliveira Barcaui: Approval of the final version of the manuscript; conception and planning of the study; elaboration and writing of the manuscript; obtaining, analysis, and interpretation of the data; effective participation in research orientation; intellectual participation in the propaedeutic and/or therapeutic conduct of the studied cases; critical review of the literature; critical review of the manuscript.

Juan Piñeiro-Maceira: Approval of the final version of the manuscript; conception and planning of the study; elaboration and writing of the manuscript; obtaining, analysis, and interpretation of the data; effective participation in research orientation; intellectual participation in the propaedeutic and/or therapeutic conduct of the studied cases; critical review of the literature; critical review of the manuscript.

## Conflicts of interest

None declared.
